# Increased psychological distress among young people before and during the fifth wave of COVID-19 after two years of pandemic in Hong Kong: a 6-month longitudinal study

**DOI:** 10.1186/s12888-023-04933-3

**Published:** 2023-06-15

**Authors:** Stephanie MY Wong, Eric YH Chen, YN Suen, Winky Ho, Sherry KW Chan, Edwin HM Lee, KT Chan, Simon SY Lui, Michael TH Wong, Christy LM Hui

**Affiliations:** 1 Department of Psychiatry, School of Clinical Medicine, LKS Faculty of Medicine, The University of Hong Kong, Pokfulam Road, Hong Kong Hong Kong; 2 The State Key Laboratory of Brain and Cognitive Sciences, The University of Hong Kong, Pokfulam Road, Hong Kong

**Keywords:** Youth mental health, COVID-19, Omicron, Smartphone overuse, Physical activity

## Abstract

**Background:**

Despite over two years of COVID-19 worldwide, the outbreak of the Omicron variant has given rise to an unprecedented surge of infection with diverse lockdown measures implemented globally. Whether the emergence of a new wave of COVID-19 could further affect mental health in the population after nearly two years of the pandemic remains to be addressed. Furthermore, whether changes in smartphone overuse behaviours and physical activity – both of which are particularly relevant to young people – would together contribute to changes in distress symptoms during this wave of COVID-19 was also examined.

**Methods:**

A total of 248 young people from an ongoing household-based epidemiological study in Hong Kong who completed their baseline assessments prior to the Omicron variant outbreak, i.e., fifth wave of COVID-19 (July–November 2021), were invited for a 6-month follow-up study during this wave of infection (January–April 2022) (mean age = 19.7 years, SD = 2.7; 58.9% females). At both time points, levels of global distress symptoms, perceived stress, smartphone overuse, frequency of engagement in vigorous physical activity, and other potential risk and protective factors were assessed.

**Results:**

The proportion of young people presenting moderate-to-severe distress (6-item Kessler Psychological Distress Scale ≥ 5) significantly increased from 45.6 to 54.4% during the fifth wave of COVID-19 (*p* < 0.010). Significantly increased levels of smartphone overuse and reduced days of vigorous physical activity were also observed during the fifth wave. Notably, increased smartphone overuse and reduced physical activity both additively and interactively contributed to elevated distress at 6 months, even after accounting for demographic characteristics, psychiatric history, childhood adversity, as well as baseline distress symptoms, resilience, and recent personal stressors.

**Conclusions:**

The findings suggest that the emergence of a new wave of COVID-19, specifically the Omicron outbreak, can further aggravate mental distress even after a protracted period of the pandemic. Awareness of the dynamic nature of COVID-19 is necessitated to address the pressing mental health needs of populations. Supporting young people in healthier patterns of smartphone use and physical activity can be helpful.

**Supplementary Information:**

The online version contains supplementary material available at 10.1186/s12888-023-04933-3.

## Background

For over two years, the global society has experienced multiple waves of COVID-19 infections since its initial outbreak with new variants of concern. The Omicron variant, despite its possibly lower case fatality, has raised serious public health concerns owing to its higher transmissibility and lower vaccine efficiency [1–3]. Notably, the Omicron ​​variant had emerged at a time of prolonged COVID-19 stress [4, 5]. Whether individuals will adapt and become acclimatised to pandemic lockdowns, or rather become sensitised amid the ongoing fluctuations of COVID-19, becomes an ever-important question.

Studies across countries have raised concerns over the mental health impacts of COVID-19 on the general population, such as increased levels of depressive and anxiety symptoms, as well as psychological distress [5–9]. Of note, the increase in mental distress in relation to COVID-19 appeared most pronounced among young people [6, 10]. Few studies, however, have yet examined factors that can potentially be modified during lockdowns to prevent the negative mental health impacts of COVID-19 [11].

Based on observations from earlier studies, the World Health Organization (WHO) has published several reports to consolidate existing findings on the impact of COVID-19 as a guide for further research [12–14]. The barriers to access to traditional mental health services had also been also highlighted, together with suggestions on approaches that may be adopted during this period, such as mental health helplines, outreach services, teletherapy, as well as self-help techniques [12–14].

Specifically, maintaining regular physical activity and staying connected with others (e.g., via phone or online), while minimising the reading of distress-inducing pandemic news, have been raised to be helpful strategies in promoting mental well-being during COVID-19 [13]. Indeed, the mental health benefits of regular physical activity (e.g., maintaining vigorous-intensity aerobic and muscle-strengthening activities at least three days a week for adolescents [15]) have been shown in prior work [16–19]. Alongside physical activity, with the growing reliance on digital devices today, smartphones have become a convenient means for young people to maintain social relationships and connections with their peers. Nevertheless, unmonitored and prolonged uses of smartphones can increase the risk of smartphone overuse (also referred to as smartphone addiction and compulsive or excessive smartphone use), which has been associated with symptoms of depression, anxiety, and psychological distress [20–23]. A recent study in an epidemiological sample of young people in Hong Kong has further demonstrated the longitudinal impact of smartphone overuse on severe depressive symptoms and functioning for up to 1 year [24].

Notably, several initial studies have reported significant increases both in sedentary behaviours [25, 26] and smartphone use and overuse [27, 28] in young people since the pandemic. Given that both these factors are modifiable (i.e., may be targets of interventions) and are generally less stigmatising, a more in-depth study into how these factors may together contribute to changes in mental health amid the Omicron lockdown period may offer new insights into the design of more effective and youth-friendly interventions for young people.

Existing evidence in the literature, however, is mostly cross-sectional, with data collected during the initial outbreak of the pandemic; the investigation of *changes* in distress over time as new waves of infection emerge has seldom been reported. Among the several longitudinal studies available, increased levels of mental distress and psychiatric symptoms have been reported during the early months of COVID-19 [6, 10, 29, 30]. For instance, a representative household-based longitudinal study in the United Kingdom found a significant increase both in the prevalence of severe psychological distress (from 18.9 to 27.3%) and mean levels of distress (from 11.5 to 12.6) compared to the pre-COVID period (2018–2019) [6]. Interestingly, several studies have in contrast observed a gradual decline in distress across waves of COVID-19 [31, 32], which have been taken to suggest a potential “habituation” effect. However, to the best of our knowledge, all such studies had been conducted before the Omicron outbreak [6, 10, 29]. With the distinct profile of the Omicron variant, whether mental distress of the population can be further aggravated after nearly two years of protracted COVID-19 remains to be explored.

Compared to other populations, the COVID-19 situation had generally been well-contained in Hong Kong. This was until the surge of the Omicron variant in December 2021, which resulted in the most severe wave of infection thus far [33, 34]. By March 2022, the 7-day average of new death rates related to COVID-19 had reached one of the highest in the world [35, 36]. While restrictions began to be gradually lifted in other countries, the local government has implemented some of the most restrictive lockdown measures to date [34], including city-wide dine-in restrictions, school lockdowns, work-from-home policies, strict travel restrictions (e.g., flight bans), as well as compulsory COVID-19 testing and compulsory quarantine at designated centres. The high population density and limited living space in Hong Kong make such large-scale lockdowns considerably more difficult to cope with [37, 38]. The unprecedented outbreak of this wave of COVID-19 not only brought about immense disruptions to the everyday lives of the population, but also an immense strain on the public health system and the economy [34, 39, 40].

Of note, before the advent of COVID-19 in early 2020 in Hong Kong, the population has already experienced months of large-scale social unrest from June 2019, with city-wide protests evolving into police-civilian confrontations involving the use of tear gas, bean bag rounds, and rubber bullets [41, 42]. Arrests in connection with the social unrest have in fact been made throughout different waves of COVID-19, including the period of the Omicron outbreak in 2022 [43, 44]. Local data have shown that these two types of population-level stressors (social unrest and pandemic-related) can interact with personal stressors to increase depressive and PTSD symptoms, particularly in young people [45, 46]. A cross-cultural study has also observed disproportionately higher rates of suicidal ideation among young adults during COVID-19 in Hong Kong compared to other Asian and Western countries (e.g., the Philippines, Canada, United States, United Kingdom, New Zealand, Belgium, and Switzerland), which has been attributed to the cumulative effects of the social unrest and COVID-19 [47]. Any further increase in mental distress since the surge of the Omicron variant would therefore suggest its potential to inflict further stress in a population where distress and tension are already rife.

In view of gaps in the current literature, we conducted a 6-month longitudinal study to examine changes in psychological distress among young people from an ongoing epidemiological study before and during the fifth wave of COVID-19 and the potential factors that may contribute to such changes. We hypothesised that young people would show significantly increased distress symptoms and perceived stress during this wave of the Omicron outbreak. We also anticipated a significant increase in smartphone overuse behaviours and reduction in days of vigorous physical activity during this period, which may both additively and interactively contribute to the increased levels of distress observed among these young people.

## Methods

### Sample and study design

Young people between the ages of 15 and 24 were consecutively invited from an ongoing household-based epidemiological study of youth mental health (HK-YES) in Hong Kong. A stratified multistage cluster sampling design was adopted in the larger HK-YES to improve sample representativeness. Specifically, invitation letters were posted to a random selection of addresses obtained from the local government estimated with a residing young person within the age range (stratified by geographic district and housing type). Data were collected through face-to-face interviews by trained researchers in the HK-YES, with the option for online video conferencing following the same procedures during COVID-19. Further details of the HK-YES have been described in our prior works [37, 48, 49]. For the current follow-up study, all participants invited from the HK-YES (n = 274) agreed to take part and had their baseline assessments completed between July and November 2021 (before the Omicron variant outbreak), with 6-month follow-up assessments completed between January and April 2022 (during the Omicron outbreak). Among these participants, none explicitly refused to take part at follow-up, although we were unable to contact 23 of the participants (8.4%). Among the rest who took part in the study at both time points (n = 251), a total of 248 participants provided complete data and were included in this study (98.8%).

The current sample had a mean age of 19.7 years (SD = 2.7) and was comprised of 58.9% (n = 146) females. Seven per cent (n = 17) reported having a psychiatric history. The core demographic characteristics of this sample and the Hong Kong population are presented in Supplementary Material 1. Participants of this study were slightly older (56.0% aged 20–24 vs. 52.7% in the Hong Kong population) and more likely to be females (58.9% vs. 54.3%). Similar proportions in housing type and geographic districts between this sample and the Hong Kong population were observed.

Written informed consents were obtained from all participants or from their parents or guardians for those below the age of 18. The study was approved by the Institutional Review Board of the University of Hong Kong/Hospital Authority Hong Kong West Cluster.

### Measures

Apart from participant background information collected as part of the larger HK-YES study (e.g., age, gender, experience of childhood adversity before the age of 17), we obtained data including psychiatric history, global distress symptoms, resilience, as well as experience of personal life stressors, at before and during the fifth wave of COVID-19 through self-administered surveys. Examples of items and their reliability indices are included in Supplementary Material 1.

#### Global distress symptoms

Global distress symptoms were assessed using the 6-item Kessler Psychological Distress Scale (K6) [50]. Items were rated on a 5-point Likert scale (“all of the time” to “none of the time”) and summed to generate a composite score. The K6 has been widely adopted to identify individuals at risk for potential psychiatric disorders both before and during COVID-19, including in some longitudinal studies [51–55]. Validation of the Chinese version of the K6 has been established among young people in Hong Kong [56]. The conventional cut-off score of 5 or above was used to indicate moderate-to-severe distress symptoms [57, 58]. The internal consistency of the K6 was good in this study (α = 0.89 and α = 0.87 at baseline and follow-up, respectively).

#### Perceived stress

Perceived stress was assessed using the single-item subjective level of stress (SLS-1) [49], which directly asks participants to indicate the level of stress they consider themselves to have experienced over the past month on a scale of 0 to 10 (0=“not at all”, 10=“extremely”). The content and face validity, incremental validity, as well as predictive validity of the SLS-1 have previously been established in local epidemiological and community youth samples [49].

#### Smartphone overuse

Smartphone overuse was assessed using two items adapted from the Revised Chen Internet Addiction Scale (CIAS-R) [59] to capture (i) the compulsive use of the Internet through smartphones (“I feel uneasy and am unable to control my compulsion to use the smartphone for the Internet once I stop using it even for just a short period”) and (ii) its impact on work or school and interpersonal relationships (“Using my smartphone to go online has negatively affected my studies or work and relationship with friends or family”). Specifically, the experience of compulsive use has been noted to be one of the key features of smartphone overuse and addiction [60, 61]. The first item used in this study was also based on those of the CIAS-R (11 “fail to control the impulse” and 2 “feel uneasy once I stop going online”) that contributed more strongly to the internal consistency of the scale using data from the larger representative youth epidemiological sample by our team (see Supplementary Material 2). Meanwhile, the second item was based on items of the CIAS-R that captures the impact of smartphone overuse on social and occupational functioning. Both items were rated on a 4-point Likert scale (“strongly disagree” to “strongly agree”). In this study, a rating of “agree” or “strongly agree” was used to define the presence of smartphone overuse and significant functional impact, respectively. Those who reported “strongly disagree” or “disagree” at baseline and “agree” or “strongly agree” at follow-up were considered to have shown increased smartphone overuse or increased functional impact during the Omicron outbreak.

#### Physical activity

Vigorous physical activity was assessed using an item from the International Physical Activity Questionnaire [62], which asks participants the number of days they have engaged in vigorous physical activities like heavy lifting, digging, aerobics, or fast bicycling during the past 7 days. Those who reported 3 days or more at baseline and less than 3 days at follow-up were considered to have shown reduced engagement in vigorous physical activity.

#### Resilience and stressful life events

Resilience was assessed using the 2-item Connor-Davidson Resilience Scale (CD-RISC-2), which includes the items “able to adapt to change” and “tend to bounce back after illness or hardship” adapted from the original 25-item CD-RISC [63]. The two items were rated on a 5-point Likert scale (“not true at all” to “true nearly all of the time”) and summed to generate a composite score. The validity and reliability of the Chinese version of the CD-RISC-2, as tested against the full CD-RISC, have been established among the general population in Hong Kong [64]. Cronbach’s alpha of the CD-RISC-2 in this study was 0.70 and 0.73 at baseline and follow-up, respectively.

Personal stressful life events (SLEs) were assessed using the Life Event Checklist for DSM-V [65], which contains 17 SLEs assessed on a binary checklist (yes/no), such as assault (physical, sexual), life-threatening illness or injury, and sudden violent death of a significant other. Items were also summed to determine the number of SLEs experienced.

#### Personal background factors

Psychiatric history was determined by asking participants whether they had received any official psychiatric diagnosis. Childhood adversity was assessed using items from the childhood section of the Composite International Diagnostic Interview 3.0 [66], which covers experiences in the domains of emotional abuse, physical abuse, neglect, and sexual abuse, with each item rated on a 5-point Likert scale (“never” to “very often”). A score of 3 or above given to any of the childhood adverse experiences was used to define the presence of childhood adversity.

### Statistical analysis

Descriptive statistics at baseline and follow-up were generated for all variables (including distress symptoms, perceived stress, resilience, and experience of SLEs). We first tested whether the proportion of participants with moderate-to-severe distress symptoms (K6 ≥ 5) would increase at follow-up during the COVID-19 surge using the McNemar test. We adopted a K6 threshold of 5 or above which corresponds to moderate-to-severe distress symptom level [57, 58] (see above). To test the hypothesis that smartphone overuse and physical activity would be related to increased distress outcomes, we conducted a series of repeated measure ANOVAs and McNemar tests to compare levels of distress symptoms, perceived stress, smartphone overuse (both compulsive use and functional impact), engagement in vigorous physical activity, as well as resilience, and experience of SLEs, before and during the fifth wave of COVID-19 in Hong Kong.

A two-way ANOVA was then applied to determine the potential additive and interaction effects of increased smartphone overuse and reduced vigorous physical activity on 6-month distress symptoms during the fifth COVID-19 wave. Potential confounders, including age, gender, psychiatric history, childhood adversity, as well as baseline K6 score, baseline resilience, and recent personal SLEs (i.e., those reported at 6 months), were accounted for as controlled variables. To further determine the implications of smartphone overuse, another set of two-way ANOVA was applied to determine the potential additive and interaction effects of increased *functional impact* of smartphone overuse and reduced vigorous physical activity on 6-month distress symptoms (see Supplementary Material 5). All analyses were performed using SPSS version 26.0, with statistical significance set at the *p* < 0.05 level.

## Results

The proportions of young people with moderate-to-severe mental distress significantly increased from 45.6% (n = 113) to 54.4% (n = 135) during the fifth wave (*p* = 0.009). Significant increases in global distress symptoms and perceived stress were also observed during the fifth wave of COVID-19 (K6: mean = 5.99, SD = 4.42 vs. mean = 5.31, SD = 4.48; SLS-1: mean = 5.62, SD = 2.02 vs. mean = 4.92, SD = 2.36), both *p* < 0.01 (Table 1).

**Table 1:** Changes in distress symptoms, perceived stress, smartphone overuse, vigorous physical activity, and other risk and protective factors before and during the fifth wave of COVID-19 among young people in Hong Kong

	**Baseline**	6-month follow-up	*p*
	(before the fifth wave)	(during the fifth wave)	
	mean (SD) / n (%)	mean (SD) / n (%)	
Distress and perceived stress			
Global distress symptoms (K6)	**5.31 (4.48)**	**5.99 (4.42)**	**0.003**
Moderate-to-severe distress (K6 ≥ 5), n (%)	**113 (45.6)**	**135 (54.4)**	**0.009**
Perceived stress (SLS-1)	**4.92 (2.36)**	**5.62 (2.02)**	**< 0.001**
Smartphone overuse^a^			
Compulsion to use and feeling of unease when withdrawn, n (%)	**104 (41.9)**	**113 (45.6)**	**< 0.001**
Impact on studies/work and interpersonal relationships, n (%)	**62 (25.0)**	**73 (29.4)**	**< 0.001**
Days of vigorous physical activity, n (%)	**91 (36.7)**	**69 (27.8)**	**< 0.001**
Other risk and protective factors			
Resilience (CD-RISC-2)	4.88 (1.42)	4.90 (1.40)	0.831
Personal SLEs (LEC)	1.25 (1.84)	1.22 (1.84)	0.815

^a^ Defined by a rating of agree or strongly agree to the symptom

Note: Values are presented in the form of mean (SD) unless otherwise specified. Statistics significant at the p < 0.05 level are in boldface. CD-RISC-2 = 2-item Connor-Davidson Resilience Scale; K6 = 6-item Kessler Psychological Distress Scale; LEC = Life Event Checklist; SLEs = personal stressful life events; SLS-1 = single-item Subjective Level of Stress measure

In addition, significantly increased rates of smartphone overuse (compulsive use: 45.6% vs. 41.9%) and functional impact of smartphone use (29.4% vs. 25.0%), as well as reduced vigorous physical activity (27.8% vs. 36.7%), were also observed, all *p* < 0.001. No significant difference was observed in the levels of resilience and the number of SLEs experienced before and during the fifth COVID-19 wave, both *p* > 0.05.

### Impact of increased smartphone overuse and reduced vigorous physical activity on distress symptoms before and during the fifth COVID-19 wave

In this sample, 15.7% (n = 39) showed increased smartphone overuse (i.e., no significant compulsive smartphone use at baseline and with compulsive use at follow-up), while 21.0% (n = 52) showed reduced vigorous physical activity (i.e., ≥ 3 days at baseline and < 3 days at follow-up) during the fifth COVID-19 wave. Descriptive statistics of these K6 distress scores are presented in Supplementary Material 4.

**Fig. 1:**
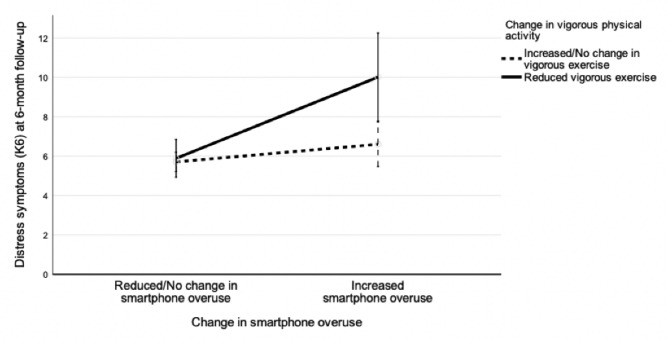
Interaction effects of increased smartphone overuse and reduced physical activity on six-month distress symptoms during the fifth wave of COVID-19 in Hong Kong. Note: K6 = 6-item Kessler Psychological Distress Scale

Using 6-month distress symptoms as the outcome, we found significant independent main effects of both increased smartphone overuse, *F*(1, 237) = 14.0, *p* < 0.001, and reduced vigorous physical activity, *F*(1, 237) = 5.6, *p* = 0.019, as well as a significant smartphone overuse X vigorous physical activity interaction effect, *F*(1, 237) = 5.2, *p* = 0.024, even after adjusting for demographics, psychiatric history, childhood adversity, baseline distress symptoms, resilience, as well as recent personal SLEs. These additive and interaction effects are illustrated in Fig. 1), with detailed findings presented in Table 2.

Similar findings were observed when considering the additive and interaction effects of increased functional impact of smartphone overuse and reduced vigorous physical activity on 6-month distress symptoms (all *p* < 0.05). Detailed findings are presented in **Table S4** of Supplementary Material 5.

**Table 2:** Additive and interaction effects of increased smartphone overuse and reduced vigorous physical activity on distress symptoms during the Omicron outbreak among young people in Hong Kong

	Distress symptoms (K6) at 6-month				
	follow-up (during the fifth wave)				
	SS	df	MS	*F*	*p*
Background factors (controlled variables)					
Age	4.57	1	4.57	0.47	0.496
Gender	3.17	1	3.17	0.32	0.571
Psychiatric history	0.09	1	0.09	0.01	0.926
Childhood adversity	24.3	1	24.3	2.48	0.117
Baseline symptoms, resilience, and recent stressors (controlled variables)					
**Distress symptom (K6) at baseline**	**1296.32**	**1**	**1296.32**	**132.05**	**< 0.001**
Resilience (CD-RISC-2) at baseline	30.4	1	30.4	3.1	0.08
**Personal SLEs (LEC) at follow-up**	**100.21**	**1**	**100.21**	**10.21**	**0.002**
Smartphone overuse and vigorous physical activity					
**Increased smartphone overuse (compulsive use)**	**137.46**	**1**	**137.46**	**14**	**< 0.001**
**Reduced vigorous physical activity**	**54.69**	**1**	**54.69**	**5.57**	**0.019**
Increased smartphone overuse (compulsive use) *	**50.83**	**1**	**50.83**	**5.18**	**0.024**
**reduced vigorous physical activity**					
Error	2326.66	237			
Total	13730	248			

*Note*. Statistics significant at the *p* < 0.05 level from the two-way ANOVA are in boldface. CD-RISC-2 = 2-item Connor-Davidson Resilience Scale; K6 = 6-item Kessler Psychological Distress Scale; LEC = Life Event Checklist; SLEs = personal stressful life events

## Discussion

To the best of our knowledge, this was among the first study to examine changes in mental health before and during the surge of the Omicron outbreak amid nearly two years of protracted COVID-19. We found that the emergence of the Omicron variant had contributed to a further increase in distress among young people on top of the prolonged pandemic. Notably, this elevation in distress was related to increased smartphone overuse and reduced physical activity not only in an additive but also multiplicative manner. Our findings highlighted the need to consider strategies to mitigate the consequences of similar public health crises and lockdowns in the future both in terms of mental health interventions and policy design.

The current work added to the literature in the discussion of whether mental health in young people will show patterns of adaptation to pandemic lockdowns or that their symptoms can become further aggravated as a new wave of COVID-19 emerges. In addition to examining changes in mental distress due to the Omicron outbreak, we were also interested in identifying potentially modifiable factors that may be involved in the process. We found that two important factors, namely smartphone overuse and reduced physical activity, had also been exacerbated and contributed to the elevated distress observed during the Omicron lockdown.

Particularly during lockdown periods where in-person activities and social contacts are restricted, it is acknowledged that smartphones can offer a convenient platform for such communications, the gratification of various needs, as well as coping with daily life stress [67, 68]. However, while frequent uses of smartphones may not necessarily be problematic, smartphone overuse can have significant impacts on mental health (also reported before COVID-19 [69, 70]) and can further contribute to poorer psychosocial functioning and both academic and work performance [24, 71]. There had also been suggestions that an increased dependence on smartphone use during COVID-19 can further perpetuate pandemic-related fears and distress [72]. The repeated cycles of news reading can possibly trigger a state of “event-based rumination” (i.e., repetitive thoughts about an external event), which has been shown to be associated with increased psychopathological symptoms amid large-scale population-level stress [73–75].

Meanwhile, although reduced physical activity may be understandable in the context of COVID-19, the impact that extended periods of lockdown restrictions has on mental health is of concern. Importantly, our previous study using the experience sampling method has found that engagement in active leisure activities can have protective effects against the impact of restrictive COVID-19 lockdown measures on momentary negative affect [37]. Offering suggestions to young people for maintaining physical activity that can be engaged in at home would be helpful (e.g., rope jumping, fast dancing or aerobic dancing, and even martial arts, are some examples of vigorous-intensity physical activities [76]).

Overall, supporting young people in the healthy use of smartphones and maintaining physical activity during COVID-19 is important. While few studies to date have systematically examined the effectiveness of interventions targeting smartphone overuse, a recent systematic review noted that a combination of psychological and behavioural interventions – particularly exercise interventions – may be optimal [77]. Together with our findings suggesting significant interactions between increased smartphone overuse (both compulsive use and its functional impacts) and reduced physical activity, incorporating exercise interventions in psychological interventions (whether delivered offline or online) may be further explored not only for maintaining physical activity, but also for preventing the worsening of smartphone overuse and improving mental health outcomes.

In the interpretation of findings of this study, we also note that some studies have reported patterns of habituation towards COVID-19 prior to the Omicron outbreak. For instance, findings from a previous local study based on data from an online text-based counselling service (N = 60,775) suggested signs of adaption and improved resilience in community members across four waves of COVID-19 from January 2019 to January 2021 [31]. Another longitudinal study in the United Kingdom has also reported a trend of reduced depressive and anxiety symptoms across the first 20 weeks of initial COVID-19 lockdown [32]. As with the majority of pandemic-related research, however, these studies had only covered the initial periods of COVID-19. Our current observations suggested a hitherto unanticipated effect: that after nearly 24 months of COVID-19, the Omicron surge was still able to trigger a significant aggravation in mental distress. This observation may at least partly be the result of fatigue following an extended period of COVID-19. The different nature of the Omicron variant (e.g., rapid spread to a large proportion of the population, extensive lockdowns in some areas) might also have played a role. These considerations highlighted the need for continued monitoring of the trajectory of youth mental health in the population across periods of COVID-19, as well as in the post-COVID period.

In addition, we did not find changes in resilience and the frequency of stressor exposure from before to during the fifth wave of COVID-19 in this sample. One of the reasons may owe to the assessment of resilience as a general trait (e.g., able to “adapt to change” and “bounce back”), which may be less susceptible to change as compared to adaptation in relation to COVID-19 or towards more severe or traumatic events. The two-year period of COVID-19 prior to the Omicron outbreak may also have equipped the population in adapting to the pandemic. Indeed, more recent studies have emphasised resilience not as the absence of psychopathology, but rather a dynamic and context-dependent process influenced by interdependent systems of developmental, neurobiological, psychological, and environmental factors [78, 79]. Identifying whether subgroups of individuals may be resilient (or more vulnerable) to the impact of COVID-19 lockdowns may facilitate the identification of young people at greater mental health risks. In addition, while an “others” option was provided in the LEC for assessing personal stressors, the stressful events covered in the LEC may be less sensitive to everyday life stressors, which may be more common during COVID-19 lockdowns. Examining the impact of pandemic-related stressors, as well as how their experiences might have differed from earlier waves of the COVID-19, may be helpful for designing more specific and context-relevant interventions.

### Strengths and limitations

In contrast to existing longitudinal and cross-sectional studies conducted during the initial phases of COVID-19, the current study provided evidence to document the mental health impacts of the Omicron outbreak. With the use of a longitudinal design, the current investigation offered insights into whether distress levels could be further exacerbated in the context of protracted periods of population stress. We also examined the potential contribution of smartphone overuse and reduced physical activity – both of which are modifiable and less stigmatising – to elevated distress in young people. The observation that these two factors can potentiate the effect of one another to affect mental health outcomes, even after accounting for a wide range of potential confounding factors, suggested the potential importance of considering their roles in future interventions.

Nonetheless, we also acknowledge some limitations in the study. While the K6 is a widely validated measure and has shown to be valid in predicting risk for psychiatric disorders, it cannot replace clinical diagnosis. Our current findings should be taken as a reference for the *change* in those meeting the threshold of moderate-to-severe distress and distress severity instead of prevalence data. Due to the need for timely data collection, the use of simple self-administered measures is a common practice for tracking the differences in mental health before and since the outbreak of COVID-19 [6, 10, 29, 30]. Studies that aim to establish changes in the prevalence of diagnosable conditions may combine both self-administered and clinician-rated data in the future. Despite the satisfactory follow-up rate and similar profiles of this sample and the Hong Kong population in terms of housing type and household geographic district, our sample was comprised of slightly older and female participants. If longitudinal data before and during later waves of COVID-19 were available from other studies, it would be ideal to test the current observations in other settings and populations to determine their generalisability.

In addition, brief measures indeed can be valid measures of specific phenomena and have the benefit of simple administration, which are particularly beneficial when working with young people and in time-limited settings [49, 80]. Nonetheless, other related symptoms or subdomains of a phenomenon could not be fully captured, which can possibly provide additional information for specific intervention planning. Our single-item measures of smartphone overuse were not intended to determine clinical levels of smartphone addiction but rather to capture two key aspects of the phenomenon, namely compulsion and its impact on social and occupational functioning. It would be worthwhile to further examine whether other symptoms of smartphone overuse, such as tolerance and withdrawal, would also show similar patterns of associations. Lastly, while we focused on vigorous physical activity in this study, capturing physical activities also at light and moderate levels in the future may be helpful for determining the level of physical activity most beneficial for mental health in young people.

## Conclusion

The present study underlined the need for the continued monitoring of mental health conditions and its related outcomes in the population over time amid large-scale stressors, such as COVID-19 – especially in young people, even when the situation initially appears to be well-contained. The complexity and unpredictability of the situation prevent the absolute knowledge of whether and when a next wave of infection or other public health crises will arrive. Improving future policies based on prior empirical evidence and preparing the population with adequate strategies using timely data to combat mental health challenges for future changes are imperative.

## Electronic supplementary material

Below is the link to the electronic supplementary material.

Supplementary Material 1

## Data Availability

The data presented in the current manuscript could be made available upon reasonable request. Enquiries may be submitted to the corresponding author.
