# Supplementary material for: Increased psychological distress among young people before and during the fifth wave of COVID-19 after two years of pandemic in Hong Kong: a 6-month longitudinal study
Source: BMC Psychiatry. 2023 Jun 15;23:433. doi: 10.1186/s12888-023-04933-3 (PMC10267546; doi:10.1186/s12888-023-04933-3)
Supplement: Supplementary file 1 — Supplementary Material 1 [file 12888_2023_4933_MOESM1_ESM.docx]

Supplementary Material

**Increased psychological distress among young people before and during the fifth wave of COVID-19 after two years of pandemic in Hong Kong: a 6-month longitudinal study**

Stephanie MY Wong^1#^, Eric YH Chen^1,2^*, YN Suen^1^, Winky Ho^1^, Sherry KW Chan^1,2^, Edwin HM Lee^1^, KT Chan^1^, Simon SY Lui^1^, Michael TH Wong^1^, Christy LM Hui^1^

^#^Co-first authors

*Corresponding author

^1^Department of Psychiatry, School of Clinical Medicine, LKS Faculty of Medicine, The University of Hong Kong, Hong Kong

^2^The State Key Laboratory of Brain and Cognitive Sciences, The University of Hong Kong, Hong Kong

**Corresponding author**

**Eric Y. H. Chen**

MA(Oxon), MBChB(Edin), MD(Edin), FRCPsych, FHKAM(Psychiatry)

Chair Professor, Department of Psychiatry, The University of Hong Kong

Address: Department of Psychiatry, School of Clinical Medicine, LKS Faculty of Medicine, The University of Hong Kong, Hong Kong,

2/F New Clinical Building, Queen Mary Hospital, Pokfulam Road, Hong Kong

Email: eyhchen.hk@gmail.com

Tel: (852) 2255 4488

## Supplementary Material 1.

### Table S1 Core demographic characteristics of the current sample and the Hong Kong population

|  |  |  | **Follow-up study sample (n = 248)** | |  | **Hong Kong**  **population^a^** |
| --- | --- | --- | --- | --- | --- | --- |
|  |  |  | n | % |  | % |
| **Age** | |  |  |  |  |  |
|  | 15–19 |  | 109 | 44.0 |  | 47.3 |
|  | 20–24 |  | 139 | 56.0 |  | 52.7 |
| **Sex** | |  |  |  |  |  |
|  | Male |  | 102 | 41.1 |  | 45.7 |
|  | Female |  | 146 | 58.9 |  | 54.3 |
| **Housing type** | |  |  |  |  |  |
|  | Public rental housing |  | 80 | 32.3 |  | 30.2 |
|  | Non-public rental housing |  | 166 | 67.0 |  | 69.1 |
|  | Temporary housing / Care homes |  | 2 | 0.8 |  | 0.8 |
| **Geographic district** | |  |  |  |  |  |
|  | Hong Kong Islands |  | 31 | 12.5 |  | 13.8 |
|  | Kowloon |  | 81 | 32.7 |  | 31.1 |
|  | New Territories |  | 136 | 54.8 |  | 55.2 |
| ^a^ Data of the Hong Kong population are from on the latest 2022 Hong Kong population Census statistics based on the 15–24 age range for age and geographic districts and all ages for sex and housing type. | | | | | | |

## Supplementary Material 2.

**Measures used in the present study**

**Global distress symptoms**

The 6-item Kessler Psychological Distress Scale (Kessler et al., 2003):

| - During the last 30 days, about how often did you…  1. …feel nervous? 2. …feel hopeless? 3. …feel restless or fidgety? 4. …feel so depressed that nothing could cheer you up? 5. …feel that everything was an effort? 6. …feel worthless? |  | 0 = None of the time  1 = A little of the time  2 = Some of the time  3 = Most of the time  4 = All of the time |
| --- | --- | --- |

The K6 has been widely adopted in studies examining distress severity and its utility in determining risk for mental disorders (e.g., Green et al., 2010; Mewton et al., 2016). For instance, a study in Canada reported high internal reliability of the K6 (α=0.86) in a large epidemiological sample of young people aged 15–19 years (n = 2010) (Ferro, 2019). In this sample, K6 scores were found to be significantly associated with past-year major depressive episode, generalised anxiety disorder, and bipolar disorder according to the WHO Composite International Diagnostic Interview 3.0 (AUC = 0.847–0.853). In addition, the measurement invariance of the K6 was supported both in this youth sample (15–19 years) and in adults aged 20–64 years in the same epidemiological study (n = 2010) (Ferro, 2019). The K6 has also been utilised and validated in youth samples in Hong Kong and in mainland China (Chan & Fung 2014; Kang et al., 2015; Wong et al., 2021).

**Perceived stress**

The single-item subjective level of stress (Wong et al., 2022):

| - “Please indicate the level of stress which you consider you have experienced in the past one month on a scale of 0 to 10.” |  | 0 = Not at all  5 = Moderate  10 = Extremely |
| --- | --- | --- |

The SLS-1 was recently developed and validated using data from a large epidemiological sample of young people in Hong Kong, with its predictive validity further tested in a separate longitudinal community youth sample in Hong Kong (Wong et al., 2021).

**Smartphone overuse**

Two items adapted from the Revised Chen Internet Addiction Scale (Chen et al., 2003):

| - “I feel uneasy and am unable to control my compulsion to use the smartphone for the Internet once I stop using it even for just a short period.” - “Using my smartphone to go online has negatively affected my studies or work and relationship with friends or family.” |  | 1 = Strongly disagree  2 = Disagree  3 = Agree  4 = Strongly agree |
| --- | --- | --- |

In this study, the presence of smartphone overuse was defined by a rating of “agree” or “strongly” agree. Those who reported “strongly disagree” or “disagree” at baseline and “agree” or “strongly agree” at follow-up were considered to have shown increased smartphone overuse behaviour.

The CIAS-R has been validated in a sample of secondary school students in Hong Kong (mean age = 15.9 years; α=0.95 in Mak et al., 2014), including in the epidemiological youth sample in Hong Kong by our team (aged 15–24 years; α=0.92 in Wong et al., 2022).

**Vigorous physical activity**

An item from the International Physical Activity Questionnaire (Craig et al., 2003):

| - “During the last 7 days, on how many days did you do vigorous physical activities like heavy lifting, digging, aerobics, or fast bicycling?” |  | ______ days per week |
| --- | --- | --- |

Those who reported 3 days or more at baseline and less than 3 days at follow-up were considered to have shown reduced engagement in vigorous physical activity in this study.

The assessment of engagement in vigorous physical activity using the IPAQ has also been adopted and tested in samples involving young people (aged 15–17 years in Hagströmer et al., 2008; aged 19–29 years in Papathanasiou et al., 2009), including in Hong Kong (aged 15–55 years in Hong Kong in Macfarlane et al., 2007).

**Resilience**

The 2-item Connor-Davidson Resilience Scale (Vaishnavi et al., 2007; Ni et al., 2016):

| 1. “Able to adapt to change” 2. “Tend to bounce back after illness or hardship” |  | 0 = Not true at all  4 = True nearly all of  the time |
| --- | --- | --- |

The full scale and another abridged version on which the 2-item version was based (CD-RISC and CD-RISC-10) have been widely administered to young people across cultures (e.g., Cheng et al., 2020; Fu et al., 2014; Okuyama et al., 2018; Wong et al., 2021). The CD-RISC-2 has also been applied in previous studies in youth samples in Hong Kong (e.g., Lai et al., 2022).

**Personal stressful life event**

The Life Event Checklist (Weathers et al., 2013):

| 1. Natural disaster (for example, flood, hurricane, tornado, earthquake) 2. Fire or explosion 3. Transportation accident (for example, car accident, boat accident, train wreck, plane crash) 4. Serious accident at work, home, or during recreational activity 5. Exposure to toxic substance (for example, dangerous chemicals, radiation) 6. Physical assault (for example, being attacked, hit, slapped, kicked, beaten up) 7. Assault with a weapon (for example, being shot, stabbed, threatened with a knife, gun, bomb) 8. Sexual assault (rape, attempted rape, made to perform any type of sexual act through force or threat of harm) 9. Other unwanted or uncomfortable sexual experience 10. Combat or exposure to a war-zone (in the military or as a civilian) 11. Captivity (for example, being kidnapped, abducted, held hostage, prisoner of war) 12. Life-threatening illness or injury 13. Severe human suffering 14. Sudden violent death (for example, homicide, suicide) 15. Sudden accidental death 16. Serious injury, harm, or death you caused to someone else 17. Any other very stressful event or experience |  | 1 = Yes  0 = No |
| --- | --- | --- |

Items were also summed to determine the number of SLEs experienced.

The LEC has also been adopted in adolescent samples (e.g., undergraduate students aged 17 years or above in Pugach et al., 2021; aged 12–13 years and followed up for three years in Grummitt et al., 2022). Reliability of the LEC has also been demonstrated (intraclass correlation coefficients = 0.62–0.64; Pugach et al., 2021).

## Supplementary Material 3.

The item on smartphone overuse in the current study was adapted based on two items from the Revised Chen Internet Addiction Scale (CIAS-R): item 11 – “I fail to control the impulse” and item 2 – “I feel uneasy once I stop going online for a certain period of time”.

Aside from the significance of the experience of compulsion in smartphone overuse and addiction (Cha et al., 2018; Lin et al., 2014), we showed that these two items also contributed more strongly to the overall internal consistency of the full scale in the larger HK-YES epidemiological youth sample (n = 3033) as presented in our prior work (Wong et al., 2022).

### Table S2. Internal consistency of the adapted version of the Revised Chen Internet Addiction Scale for assessing smartphone overuse (n = 3033)

| Adapted items from the CIAS-R | Cronbach's Alpha if Item Deleted | Corrected Item-Total Correlation |
| --- | --- | --- |
| **11. Fail to control the impulse to go online.** | **0.920** | **0.66** |
| **2. Feel uneasy once stopped going online for a certain period.** | **0.920** | **0.61** |
| 19. Fail to control the impulse to go back online after logging off. | 0.920 | 0.62 |
| 22. Tried to spend less time online but unsuccessful. | 0.920 | 0.63 |
| 24. Spending an increased amount of time online to achieve the same satisfaction as before. | 0.920 | 0.62 |
| 3. Spending longer and longer periods of time online. | 0.921 | 0.56 |
| 6. Staying online for longer than intended. | 0.921 | 0.56 |
| 10. Feel distressed or down when stopped going online for a certain period of time. | 0.921 | 0.60 |
| **15. Negatively affected schoolwork or job performance as a result.** | **0.921** | **0.58** |
| **17. Decreased interactions with family members as a result.** | **0.921** | **0.57** |
| 18. Decreased recreational activities as a result. | 0.921 | 0.58 |
| 21. Negatively affected physical health. | 0.921 | 0.55 |
| 23. Sleeping less so that more time can be spent online. | 0.921 | 0.59 |
| 26. Tired during the day as a result of going online late at night. | 0.921 | 0.58 |
| 4. Feel restless and irritable when the Internet is disconnected or unavailable. | 0.922 | 0.51 |
| 5. Feel energised online n matter how tired I was. . | 0.922 | 0.48 |
| 7. No decrease in the time spent online despite negative impacts on relationships. | 0.922 | 0.49 |
| 9. Have increased substantially the amount of time spent online. | 0.922 | 0.48 |
| 12. Find myself going online instead of spending time with friends. | 0.922 | 0.52 |
| 13. Get backaches or other physical discomfort as a result. | 0.922 | 0.50 |
| 14. Going online is the first thought after waking up each morning. | 0.922 | 0.51 |
| 16. Feel like missing something if didn’t go online for a certain period of time. | 0.922 | 0.53 |
| 20. Life would be joyless without the Internet. | 0.922 | 0.48 |
| 25. Fail to have meals on time because of using the Internet. | 0.922 | 0.48 |
| 1. Was told more than once that I spend too much time online | 0.923 | 0.44 |
| 8. More than once having slept less than four hours due to being online. | 0.923 | 0.48 |
| Reliability of full scale: | 0.924 |  |

*Note*. Original items adapted from the CIAS-R were used but are slightly rephrased for the purpose of inclusion in this Supplementary Material.

## Supplementary Material 4.

### Distress symptoms at 6-month follow-up in relation to changes in smartphone overuse and physical activity

### Table S3. Severity of distress symptoms at follow-up (during the fifth wave of COVID-19) in relation to changes in smartphone overuse and days of vigorous physical activity

| Change in smartphone overuse (compulsive use) |  | Change in days of vigorous physical activity | |
| --- | --- | --- | --- |
|  |  | No reduction (n = 196) | Reduced (n = 52) |
| No increase in smartphone overuse (n = 209) |  | 5.74 (4.27) | 5.34 (4.19) |
| Increased smartphone overuse (n = 39) |  | 7.58 (4.29) | 8.63 (7.19) |

*Note*. All values are presented in the form of mean (SD). K6 = 6-item Kessler Psychological Distress Scale.

**Supplementary Material 5.**

**Interaction effects increased impact of smartphone overuse and reduced vigorous physical activity on 6-month distress symptoms**

Apart from examining the role of increased smartphone overuse (determined by compulsive use of smartphones as presented in the main text), we further examined the additive and interaction effects of increased *functional impact* of smartphone overuse (i.e., impact on studies/work and interpersonal relationships) and reduced vigorous physical activity on 6-month distress symptoms during the fifth wave of COVID-19 in Hong Kong. The findings revealed significant main and interaction effects of the two factors (all *p* < 0.05), even when accounting for personal demographics, psychiatric history, childhood adversity, as well as baseline distress symptoms, resilience, and recent personal stressors. **Figure S1** presents these effects, with detailed findings presented in **Table S4**. Baseline distress symptoms and recent personal SLES were also significant factors (both *p* < .05) (**Table S4**).


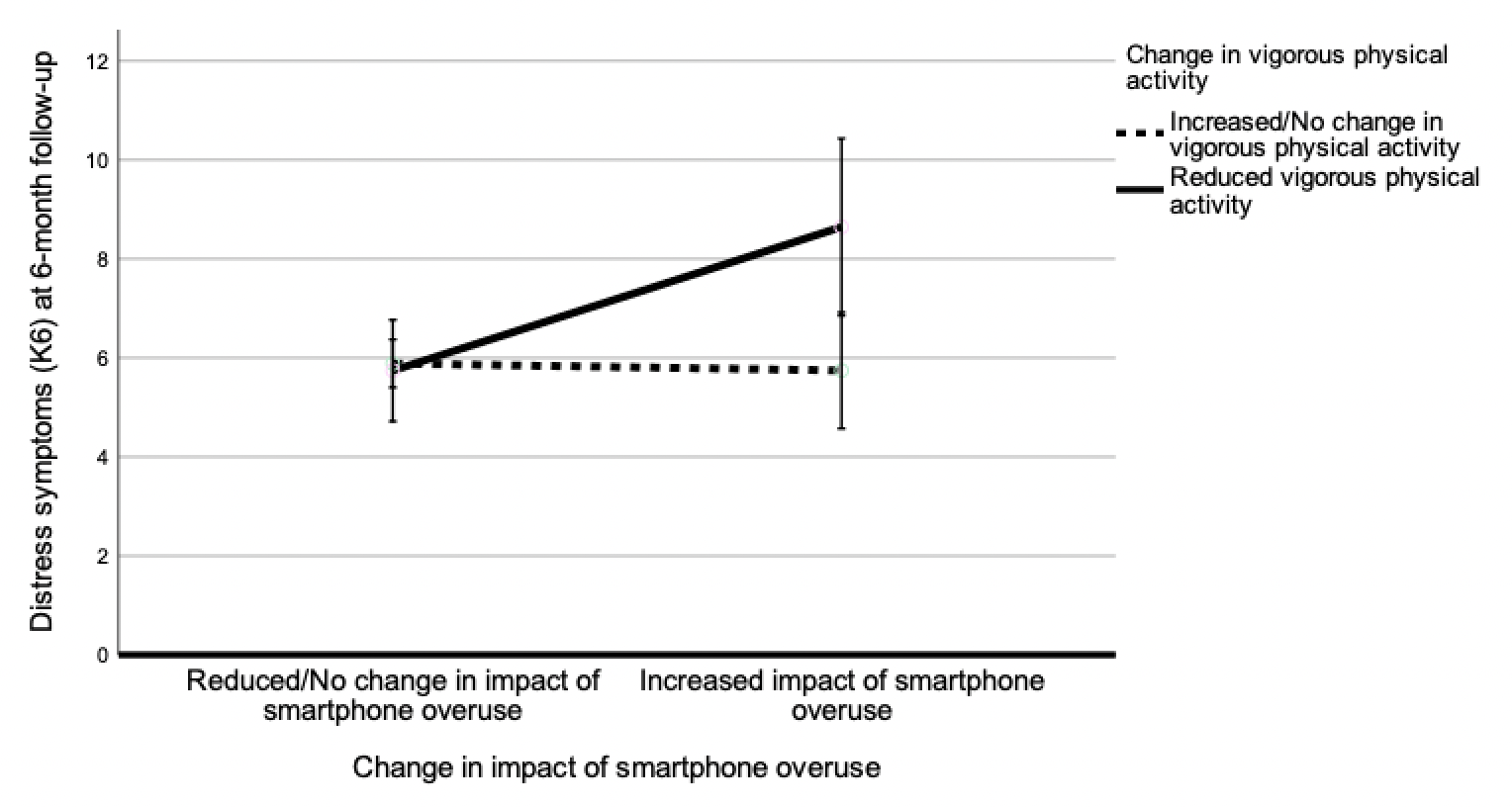


**Figure S1. Interaction effects of increased functional impact of smartphone overuse and reduced physical activity on six-month distress symptoms during the fifth wave of COVID-19 in Hong Kong**

*Note*. K6 = 6-item Kessler Psychological Distress Scale.

### Table S4. Impact of increased functional impact of smartphone overuse and reduced vigorous physical activity on six-month distress symptoms during the fifth wave of COVID-19 in Hong Kong

|  |  | Distress symptoms (K6) at 6-month  follow-up (during the fifth wave) | | | | |  |
| --- | --- | --- | --- | --- | --- | --- | --- |
|  |  | SS | df | MS | *F* | *p* |  |
| Background factors (controlled variables) | |  |  |  |  |  |  |
|  | Age | 3.49 | 1.00 | 3.49 | 0.35 | 0.556 |  |
|  | Gender | 1.96 | 1.00 | 1.96 | 0.19 | 0.660 |  |
|  | Psychiatric history | 0.17 | 1.00 | 0.17 | 0.02 | 0.896 |  |
|  | Childhood adversity | 19.11 | 1.00 | 19.11 | 1.90 | 0.169 |  |
| Baseline symptoms, resilience, and recent stressors (controlled variables) | |  |  |  |  |  |  |
|  | **Distress symptom (K6) at baseline** | **1266.57** | **1.00** | **1266.57** | **125.89** | **<0.001** |  |
|  | Resilience (CD-RISC-2) at baseline | 30.44 | 1.00 | 30.44 | 3.03 | 0.083 |  |
|  | **Personal SLEs (LEC) at follow-up** | **119.90** | **1.00** | **119.90** | **11.92** | **0.001** |  |
| Smartphone overuse and vigorous physical activity | |  |  |  |  |  |  |
|  | **Increased smartphone overuse (compulsive use)** | **52.18** | **1.00** | **52.18** | **5.19** | **0.024** |  |
|  | **Reduced vigorous physical activity** | **47.51** | **1.00** | **47.51** | **4.72** | **0.031** |  |
|  | **Increased smartphone overuse (compulsive use) ***  **reduced vigorous physical activity** | **62.66** | **1.00** | **62.66** | **6.23** | **0.013** |  |
| Error | | 2384.37 | 237.00 |  |  |  |  |
| Total | | 13730.00 | 248.00 |  |  |  |  |

*Note*. Statistics significant at the *p* < 0.05 level from the two-way ANOVA are in boldface. CD-RISC-2 = 2-item Connor-Davidson Resilience Scale; K6 = 6-item Kessler Psychological Distress Scale; LEC = Life Event Checklist; SLEs = personal stressful life events.

**References**

Cha, S. S., & Seo, B. K. (2018). Smartphone use and smartphone addiction in middle school students in Korea: Prevalence, social networking service, and game use. *Health Psychology Open, 5*(1), 1–15.

Chan, S. M., & Fung, T. C. T. (2014). Reliability and validity of K10 and K6 in screening depressive symptoms in Hong Kong adolescents. *Vulnerable Children and Youth Studies, 9*(1), 75–85.

Chen, S. H., Weng, L. J., Su, Y. J., Wu, H. M., & Yang, P. F. (2003). Development of a Chinese Internet addiction scale and its psychometric study. *Chinese Journal of Psychology, 45*(3), 279–294.

Cheng, C., Dong, D., He, J., Zhong, X., & Yao, S. (2020). Psychometric properties of the 10-item Connor–Davidson Resilience Scale (CD-RISC-10) in Chinese undergraduates and depressive patients. *Journal of Affective Disorders, 261*, 211–220.

Craig, C. L., Marshall, A. L., Sjöström, M., Bauman, A. E., Booth, M. L., Ainsworth, B. E., ... & Oja, P. (2003). International physical activity questionnaire: 12-country reliability and validity. *Medicine and Science in Sports and Exercise, 35*(8), 1381–1395.

Ferro, M. A. (2019). The psychometric properties of the Kessler Psychological Distress Scale (K6) in an epidemiological sample of Canadian youth. *The Canadian Journal of Psychiatry, 64*(9), 647–657.

Fu, C., Leoutsakos, J. M., & Underwood, C. (2014). An examination of resilience cross-culturally in child and adolescent survivors of the 2008 China earthquake using the Connor–Davidson Resilience Scale (CD-RISC). *Journal of Affective Disorders, 155*, 149–153.

Green, J. G., Gruber, M. J., Sampson, N. A., Zaslavsky, A. M., & Kessler, R. C. (2010). Improving the K6 short scale to predict serious emotional disturbance in adolescents in the USA. *International Journal of Methods in Psychiatric Research, 19*(S1), 23–35.

Grummitt, L., Barrett, E., Kelly, E. V., Stapinski, L., & Newton, N. (2022). Personality as a possible intervention target to prevent traumatic events in adolescence. *Behavioral Sciences, 12*(4), 90.

Hagströmer, M., Bergman, P., De Bourdeaudhuij, I., Ortega, F. B., Ruiz, J. R., Manios, Y., ... & Sjöström, M. (2008). Concurrent validity of a modified version of the International Physical Activity Questionnaire (IPAQ-A) in European adolescents: The HELENA Study. *International Journal of Obesity, 32*(5), S42–S48.

Kang, Y. K., Guo, W. J., Xu, H., Chen, Y. H., Li, X. J., Tan, Z. P., ... & Li, T. (2015). The 6-item Kessler psychological distress scale to survey serious mental illness among Chinese undergraduates: Psychometric properties and prevalence estimate. *Comprehensive Psychiatry, 63*, 105–112.

Kessler, R. C., Barker, P. R., Colpe, L. J., Epstein, J. F., Gfroerer, J. C., Hiripi, E., ... & Zaslavsky, A. M. (2003). Screening for serious mental illness in the general population. *Archives of General Psychiatry, 60*(2), 184–189.

Lai, A. Y., Cheung, G. O., Choi, A. C., Wang, M. P., Chan, P. S., Lam, A. H., ... & Lam, T. H. (2022). Mental Health, Support System, and Perceived Usefulness of Support in University Students in Hong Kong Amidst COVID-19 Pandemic: A Mixed-Method Survey. *International Journal of Environmental Research and Public Health, 19*(19), 12931.

Lin, Y. H., Chang, L. R., Lee, Y. H., Tseng, H. W., Kuo, T. B., & Chen, S. H. (2014). Development and validation of the Smartphone Addiction Inventory (SPAI). *PloS One, 9*(6), e98312.

Macfarlane, D. J., Lee, C. C., Ho, E. Y., Chan, K. L., & Chan, D. T. (2007). Reliability and validity of the Chinese version of IPAQ (short, last 7 days). *Journal of Science and Medicine in Sport, 10*(1), 45–51.

Mak, K. K., Lai, C. M., Ko, C. H., Chou, C., Kim, D. I., Watanabe, H., & Ho, R. (2014). Psychometric properties of the revised chen internet addiction scale (CIAS-R) in Chinese adolescents. *Journal of Abnormal Child Psychology, 42*(7), 1237–1245.

Mewton, L., Kessler, R. C., Slade, T., Hobbs, M. J., Brownhill, L., Birrell, L., ... & Andrews, G. (2016). The psychometric properties of the Kessler Psychological Distress Scale (K6) in a general population sample of adolescents. *Psychological Assessment, 28*(10), 1232–1242.

Ni, M. Y., Li, T. K., Yu, N. X., Pang, H., Chan, B. H., Leung, G. M., & Stewart, S. M. (2016). Normative data and psychometric properties of the Connor–Davidson Resilience Scale (CD-RISC) and the abbreviated version (CD-RISC2) among the general population in Hong Kong. *Quality of Life Research, 25*(1), 111–116.

Okuyama, J., Funakoshi, S., Tomita, H., Yamaguchi, T., & Matsuoka, H. (2018). Longitudinal characteristics of resilience among adolescents: a high school student cohort study to assess the psychological impact of the Great East Japan Earthquake. *Psychiatry and Clinical Neurosciences, 72*(11), 821–835.

Papathanasiou, G., Georgoudis, G. E. O. R. G. E., Papandreou, M., Spyropoulos, P., Georgakopoulos, D., Kalfakakou, V., & Evangelou, A. (2009). Reliability measures of the short International Physical Activity Questionnaire (IPAQ) in Greek young adults. *Hellenic Journal of Cardiology, 50*(4), 283–294.

Pugach, C. P., Nomamiukor, F. O., Gay, N. G., & Wisco, B. E. (2021). Temporal Stability of Self-Reported Trauma Exposure on the Life Events Checklist for DSM-5. *Journal of Traumatic Stress, 34*(1), 248–256.

Weathers, F.W., Blake, D.D., Schnurr, P.P., Kaloupek, D.G., Marx, B.P., & Keane, T.M. (2013). The Life Events Checklist for DSM-5 (LEC-5). Instrument available from the National Center for PTSD at [www.ptsd.va.gov](http://www.ptsd.va.gov)

Wong, S. M. Y., Lam, B. Y. H., Wong, C. S. M., Lee, H. P. Y., Wong, G. H. Y., Lui, S. S. Y., ... & Chen, E. Y. H. (2021). Measuring subjective stress among young people in Hong Kong: validation and predictive utility of the single-item subjective level of stress (SLS-1) in epidemiological and longitudinal community samples. *Epidemiology and Psychiatric Sciences, 30*, e61.

Wong, S. M. Y., Chen, E. Y. H., Wong, C. S. M., Suen, Y. N., Chan, D. L. K., Tsang, S. H. S., ... & Hui, C. L. M. (2022). Impact of smartphone overuse on 1-year severe depressive symptoms and momentary negative affect: Longitudinal and experience sampling findings from a representative epidemiological youth sample in Hong Kong. *Psychiatry Research, 318*, 114939.

Vaishnavi, S., Connor, K., & Davidson, J. R. (2007). An abbreviated version of the Connor-Davidson Resilience Scale (CD-RISC), the CD-RISC2: Psychometric properties and applications in psychopharmacological trials. *Psychiatry Research, 152*(2-3), 293–297.
